# A randomized, open-label, parallel pilot study investigating metabolic product kinetics of the novel ketone ester, bis-hexanoyl (R)-1,3-butanediol, over one week of ingestion in healthy adults

**DOI:** 10.3389/fphys.2023.1196535

**Published:** 2023-06-22

**Authors:** Eunice Mah, Traci M. Blonquist, Valerie N. Kaden, Dawn Beckman, Amy C. Boileau, Joshua C. Anthony, Brianna J. Stubbs

**Affiliations:** ^1^ Biofortis, Mérieux NutriSciences, Addison, IL, United States; ^2^ BHB Therapeutics Ltd., Dublin, Ireland; ^3^ Buck Institute for Research on Aging, Novato, CA, United States

**Keywords:** ketones, ketone ester, ketone di-ester, exogenous ketone, beta-hydroxybutyrate (BHB)

## Abstract

**Introduction:** Bis-hexanoyl (R)-1,3-butanediol (BH-BD) is a novel ketone ester that, when consumed, is hydrolyzed into hexanoic acid (HEX) and (R)-1,3-butanediol (BDO) which are subsequently metabolized into beta-hydroxybutyrate (BHB).

**Methods:** We undertook a randomized, parallel, open-label study in healthy adults (*n* = 33) to elucidate blood BHB, HEX and BDO concentrations for 8 h following consumption of three different serving sizes (SS) of BH-BD (12.5, 25 and 50 g/day) before (Day 0) and after 7 days of daily BH-BD consumption (Day 7).

**Results:** Maximal concentration and area under the curve of all metabolites increased proportionally to SS and were greatest for BHB followed by BDO then HEX on both Day 0 and 7. Metabolite half-life tended to decrease with increasing SS for BHB and HEX. Time to peak concentration increased with increasing SS for BHB and BDO on both days. *In vitro* incubation of BH-BD in human plasma demonstrated BH-BD undergoes rapid spontaneous hydrolysis.

**Conclusion:** These results demonstrate that orally ingested BH-BD is hydrolyzed into products that appear in the plasma and undergo conversion to BHB in a SS dependent manner, and that metabolism of BH-BD neither becomes saturated at serving sizes up to 50 g nor displays consistent adaptation after 7 days of daily consumption.

## 1 Introduction

In human metabolism, the term “ketone bodies” (ketones), refers to beta-hydroxybutyrate (BHB), acetoacetate, and acetone. Ketone bodies are lipid-derived endogenous metabolites with two central functions. First, ketones are a substrate that allow conversion of energy stored in lipids into a form that is readily oxidized by the brain, heart and other peripheral tissues, to provide a critical alternative fuel during starvation (Cahill, 2006). Second, ketones function as a signal in the molecular network that links the nutrient-environment to cellular function to regulate health-span and physiology (Newman and Verdin, 2017). The state of ketosis is commonly characterized by capillary blood BHB concentrations **≥** 0.5 mM (Mitchell et al., 1995; Laffel, 1999; Hallberg et al., 2018; Norgren et al., 2020). Physiological ketosis occurs when dietary carbohydrate intake is severely limited, such as during starvation, voluntary fasting (Cahill, 1970), or when following a very low-carbohydrate, high-fat (ketogenic) diet (Hallberg et al., 2018). Physiological ketosis is distinct from pathological ketoacidosis (blood BHB >10 mM) which is a medical emergency that occurs in dysregulated metabolic states such as alcoholic crisis or type 1 diabetes (Laffel, 1999).

Exogenous sources of ketones, such as ketone esters, are of interest as their ingestion can elevate blood ketone concentrations without the need for changes in dietary macronutrient intake to trigger endogenous ketone production. Even in the absence of starvation, ketones from exogenous sources still provide additional energy to support body function and may still act on the same “health-span promoting” signaling pathways that they modulate during starvation. In order to explore the applications of exogenous ketosis, studies have investigated ketone esters in manifold states of health and disease, including physical and cognitive performance (Cox et al., 2016; Evans and Egan, 2018; Evans et al., 2019; Poffe et al., 2019; Mujica-Parodi et al., 2020; Coleman et al., 2021; Poffe et al., 2021), blood glucose regulation (Walsh et al., 2021; Myette-Cote et al., 2018; Myette-Côté et al., 2019; Greaves et al., 2021) and cardiac function (Monzo et al., 2021; Selvaraj et al., 2022). It is hypothesized that consumption of exogenous ketones may replicate some of the beneficial effects of endogenous ketosis (Poff et al., 2020). Although several pre-clinical studies have compared ketogenic diet consumption to exogenous ketone administration (Youm et al., 2015; Cheng et al., 2019; Ang et al., 2020; Koronowski et al., 2021), the extent of the similarities between exogenous and endogenous ketosis in humans is largely unknown.

**FIGURE 1:**
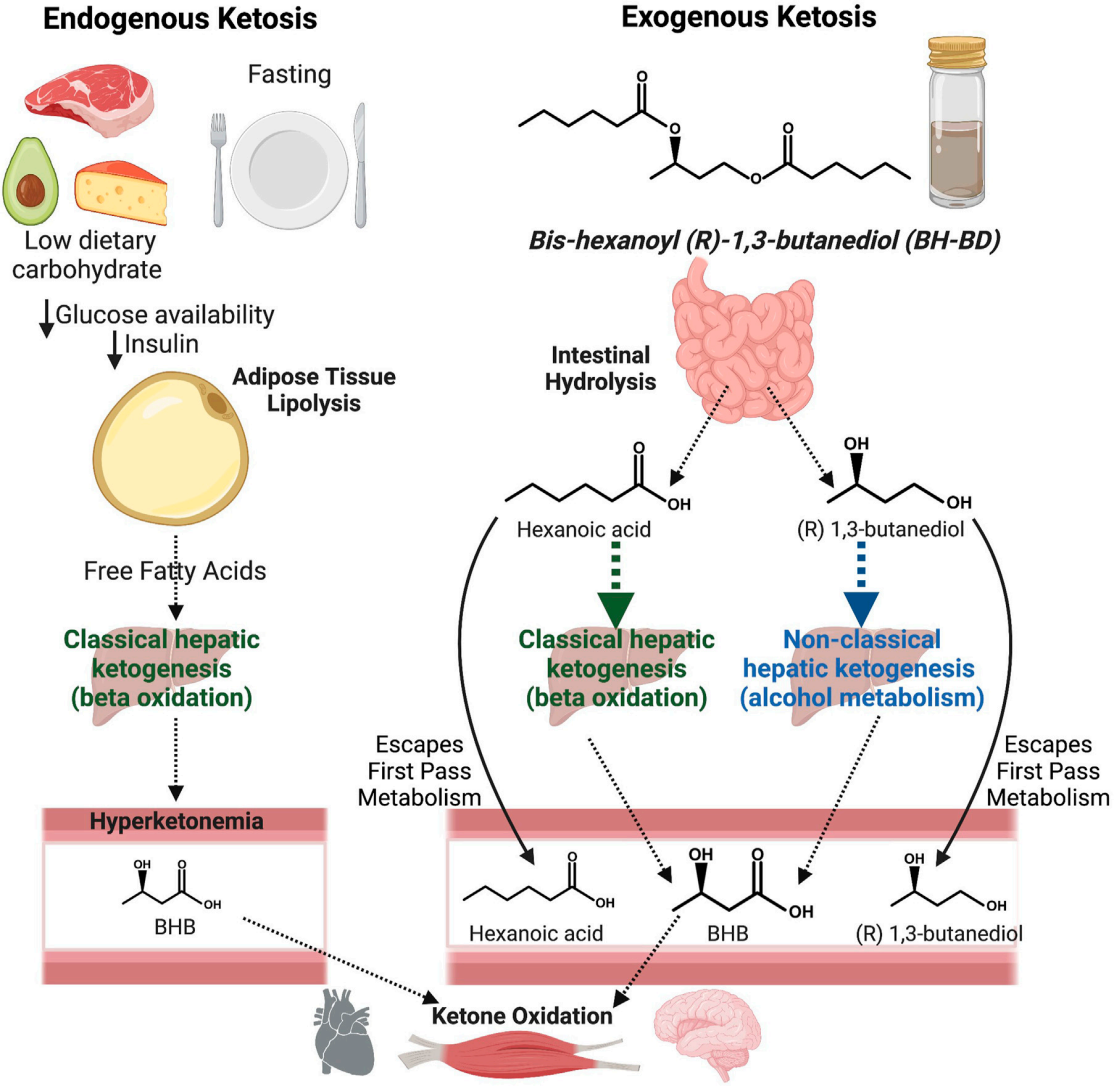
Schematic illustrating the process of endogenous ketosis resulting from diet (left side) compared to exogenous ketosis with bis-hexanoyl (R)-1,3-butanediol (BH-BD). BH-BD is hydrolyzed into hexanoic acid and (R)-1,3-butanediol, which are either metabolized into beta hydroxybutyrate (BHB) in the liver or escape first pass metabolism and are released into the systemic circulation. BHB from endogenous and exogenous ketosis are readily oxidized in tissues such as heart, skeletal muscle and brain. Figure created using BioRender.com.

Bis-hexanoyl (R)-1,3-butanediol (BH-BD, common name: C6 ketone di-ester) is a novel ketone ester that induces ketosis independently of dietary carbohydrate intake or circulating insulin concentrations. Previous *in vitro* hydrolysis studies in simulated gastric and intestinal fluids, cecal contents, fresh rat plasma and rat and dog liver microsomes informed the presumed metabolic fate of BH-BD, indicating that it is rapidly metabolized by enzymes in the small intestine or in plasma to form ketogenic precursors: hexanoic acid (HEX) - a medium chain fatty acid, and (R)-1,3-butanediol (BDO) - a ketogenic alcohol (Stubbs et al., 2021). Intact BH-BD was undetectable in plasma at all doses studied in oral gavage studies in rats (Stubbs et al., 2021). HEX and BDO are transported to the liver via the portal circulation where HEX acts as a constitutive substrate for ‘classical’ hepatic ketogenesis resulting in release of BHB and acetoacetate (Traul et al., 2000), and BDO is converted to BHB via a ‘non-classical’ hepatic ketogenic pathway (Miller and Dymsza, 1967; Mehlman et al., 1971) (Figure 1). A notable limitation of these *in vitro* studies was the lack of confirmatory human model systems. Whilst acute oral administration of BH-BD in rats, mice, and humans leads to rapid increases in blood BHB concentrations that remain elevated for several hours (Stubbs et al., 2021; Chen et al., 2021; Crabtree et al., 2022), interspecies metabolic differences clearly exist. A larger BH-BD dose was required to achieve similar blood BHB in rodents compared to humans, ∼300 mg/kg of BH-BD resulted in a BHB C_max_ of 0.84 mM in humans (Crabtree et al., 2022), whereas 1,500 mg/kg was required for a near identical BHB C_max_ of 0.84 mM in rats (Stubbs et al., 2021). Determination of the rate of BH-BD hydrolysis in a human *in vitro* model system could help to explain the intra species differences in BHB responses.

Previous work by Crabtree et al., (2022), demonstrated that a relatively small amount of HEX and BDO escapes first pass metabolism following ingestion of BH-BD, leading to an increase in the systemic concentration of both after acute ingestion of 25 g of BH-BD by healthy adults (*n* = 8). However, limited data exists on the pharmacokinetics (PK) of the hydrolysis products HEX and BDO in the systemic circulation after acute BH-BD ingestion at other serving sizes. Furthermore, whilst work using other ketone ester molecules indicates that BHB PK are unchanged after longer term ingestion, it is unknown if BH-BD metabolism and downstream metabolite PK changes after prolonged consumption.

To address these questions, we first assessed the *in vitro* hydrolysis of BH-BD in human plasma. We then undertook a randomized, open-label, parallel group pilot study in healthy adults with the objective to determine blood BHB, HEX and BDO pharmacokinetics after ingestion of one of three serving sizes (SS) of BH-BD (12.5, 25 and 50 g/day) in both a naïve state (Day 0) and after 7 days of daily BH-BD consumption (Day 7). We hypothesized that BH-BD consumption would increase BHB concentrations to the greatest extent, followed by BDO then HEX, that all metabolites would increase in a dose responsive manner, returning to baseline within 8 h post-consumption, and that the PK parameters would not significantly change between Day 0 and Day 7.

## 2 Materials and methods

### 2.1 *In vitro* BH-BD hydrolysis

*In vitro* plasma incubations were conducted at Keystone Bioanalytical Inc. (North Wales, PA, United States). Human and rat plasma, previously frozen (old) and fresh (unfrozen), was obtained from BioIVT (Westbury, NY, United States). A comparison of old and fresh plasma would allow evaluation of freeze-thaw cycle effects on plasma esterases. Plasma (5 mL) was incubated with BH-BD (20 μg/mL) in a water bath at 37°C, and 50 μL aliquots were removed at 0, 5, 15, 30, 60 and 90 min after the start of incubation. The starting concentration of BH-BD (20 μg/mL) was chosen to yield concentrations of the hydrolysis products that were within the known linear range of the assay. Aliquots were mixed with 200 μL of ice-cold internal standard (BH-BD-d3; Lot no. EW35732-10-P1 synthesized by Wuxi App Tec with 99.2% purity, 2 μg/mL) mixed for ∼2 min and centrifuged at 14,000 RPM at 4°C for 5 min 100 μL of the upper phase was removed and transferred to a HPLC injection vial with 100 μL of (de-ionized) water. Analytical methods are described below.

### 2.2 Clinical study design

**FIGURE 2:**
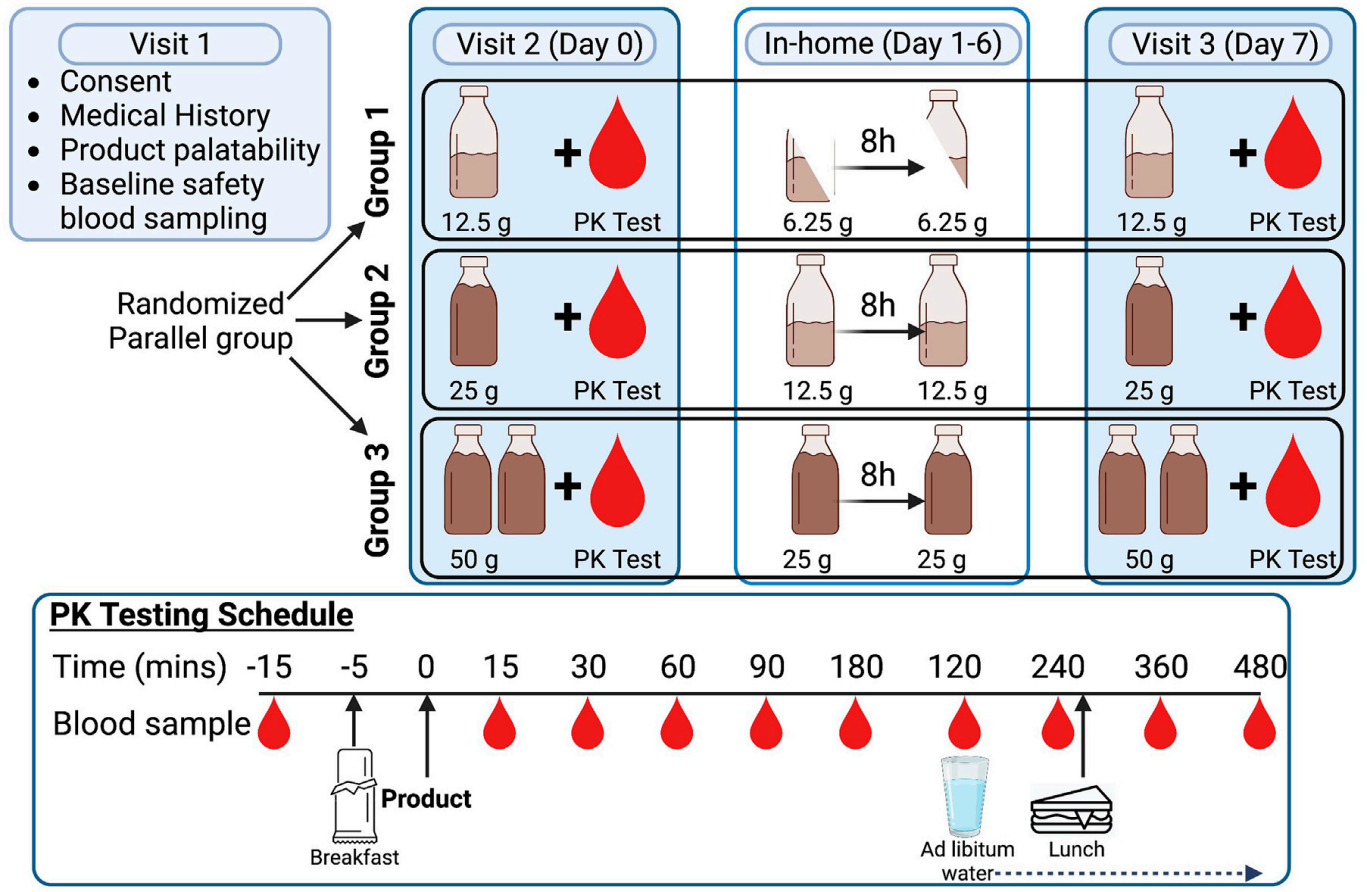
Schematic showing overall study design and schedule for pharmacokinetics (PK) visit.

Healthy adults (*n* = 33) underwent screening (Visit 1, Day −7) and took part in a randomized, open label, parallel group pilot study, that evaluated the plasma kinetic parameters of BHB, BDO and HEX for 8 h after ingestion of three SS of BH-BD in a naïve state (Visit 2, Day 0) and after 7 days of daily consumption (Visit 3, Day 7) (Figure 2). Subjects were randomly allocated to one of the BH-BD SS groups (12.5, 25 and 50 g/day), and on the Day 0 clinic visit they consumed a light breakfast and their allocated SS of study product in one bolus followed by repeated blood sampling for 8 h; a light lunch was provided 240 min after product consumption. On study Days 1—6, subjects consumed their allocated BH-BD SS at home, split into two equal servings (i.e., 7.5 g twice, 12.5 g twice or 25 g twice) spaced at least 8 h apart. On Day 7, subjects completed a clinic visit with identical BH-BD ingestion and blood sampling to the Day 0 visit.

An institutional review board (Sterling IRB, Atlanta, GA, March 15th, BIO2201) approved all study related material including the protocol and informed consent documents prior to initiation of the study. Signed informed consent and authorization for use of protected health information was provided by the subjects prior to implementation of any protocol specific procedures. The study was registered in the clinicaltrials.gov database (NCT05310058). The study was conducted in accordance with the Declaration of Helsinki and the United Sates Code of Federal Regulation Title 21. Studies took place at Biofortis Research (Addison, IL) between March 2022 and July 2022.

#### 2.2.1 Subjects and screening

Participants were healthy, aged 18—65 years, BMI 18.5–34.9 kg/m^2^. Other major exclusion criteria included: no history of major illness, no clinically important gastrointestinal conditions, not pregnant and using contraception to prevent pregnancy (females only), no recent infection, no extreme dietary habits (i.e., intermittent fasting, ketogenic diet, vegan), no unstable medication use, no recent use of weight loss supplements or medications known to influence gastrointestinal function, no recent use of ketone supplements, and no known allergies to any of the study beverage ingredients (including soy and milk protein). The full inclusion and exclusion criteria can be found in the Supplemental Information (Supplementary Table S5). At the screening visit (Visit 1, Day −7), participants completed a medical history questionnaire in addition to assessment of height, weight, BMI, vital signs, last menses (females only), current medication/supplement use, and review of inclusion/exclusion criteria to determine eligibility. Subjects were assessed for the suitability for use of an intravenous catheter by study phlebotomists. In addition, fasting (12 ± 2 h) blood samples were collected for analysis of clinical chemistry and hematology. Females under the age of 60 years completed a urine pregnancy test. Subjects had the opportunity to taste the study beverages (19 mL) followed by peanut butter to evaluate palatability.

#### 2.2.2 Study products and standard meals

BH-BD (common name: C6 ketone di-ester) was provided as an ingredient in a commercially available beverage product, “Metabolic Switch” (Juvenescence US Corp., Princeton, NJ). Commercially available Metabolic Switch was a chocolate flavored beverage, with a total volume of 75 mL per bottle, a total of 25 g BH-BD per bottle and other ingredients including: high fat whey protein, gum acacia, citric acid, soy lecithin, vegetable and fruit juice, caramel color, natural and artificial flavors, pectin, acesulfame K, sucralose, xanthan gum, sodium carboxymethyl cellulose and potassium sorbate. Each bottle contained 220 kCal, as 1 g carbohydrate, 0 g fat and 1 g protein, with the majority of calories coming from BH-BD (∼7.8 kCal/g).

The three daily SS groups were 12.5, 25 and 50 g of BH-BD, equivalent to half of one bottle, one bottle and two bottles respectively. These doses reflected the recommended serving (12.5 g BH-BD), the recommended daily maximum intake (25 g BH-BD) and 2-fold maximum intake (50 g BH-BD). At Day 0 and Day 7, study staff administered the allocated amount of BH-BD as a single serving, shaken well, chilled, consumed within 5 min and followed by 1 teaspoon of peanut butter and 8 oz water to remove the aftertaste. At home (Days 1—6) subjects were instructed to keep the study product chilled and to ensure it was well shaken before consuming as two equal daily servings each separated by 8 h. Subjects allocated to consume 12.5 g were given a 20 mL syringe and instructed to shake the bottle and use a syringe to draw up and dispense 19 mL (a quarter of a bottle) of study product into their mouth twice each day; they discarded the remaining half of the bottle at the end of the day. Those allocated to consume 25 g used a 20 mL syringe to draw up 19 mL twice (38 mL), for their first serving and consumed the remainder of the bottle for the second serving. Those allocated to 50 g consumed one whole bottle for each of their servings. Subjects recorded daily consumption in a Study Product Log, which was used to determine compliance, confirmed by subject query and by return of unused bottles on Day 7. Those who consumed 80%—120% of their allocated product were considered to be compliant.

At Day 0 and Day 7 clinic visits, subjects consumed a light standard breakfast (Nutri-Grain^®^ Softbaked Breakfast Bar) 5 min prior to study product consumption and a standard lunch following the 240 min post-product blood sample. Lunch was consumed within 30 min and subjects were instructed to eat until comfortably full on Day 0; intake was replicated on Day 7.

#### 2.2.3 Study procedures

For in-clinic testing days (Day 0 and Day 7), subjects reported to the test facility having fasted for 12 ± 2 h (water only) and having avoided exercise for 24 h. Between Days 0 and 7, subjects were asked to maintain their habitual diet and exercise habits. Subjects were queried to ensure compliance with these instructions at the start of each clinic visit. Body weight and vital signs were measured, concomitant medication and supplement use was queried, females completed a last menses questionnaire and inclusion and exclusion criteria were reviewed at the start of each visit. At Day 0, subjects were randomly allocated to one of the SS groups using a 1:1:1 sequence generated by a study statistician. An intravenous catheter was inserted into the antecubital fossa 5 min prior to the first blood sampling time point. The catheter was flushed with saline solution at least hourly during the study to maintain patency. The first blood sample was collected at t = −15 ± 10 min, where t = 0 was the time of study product consumption. Subjects consumed a light breakfast at t = - 5 min (see details above), followed by their allocated SS of study product (see details above). Following study product consumption (t = 0), blood samples were collected at 15, 30, 60, 90, 120, 180, 240, and 480 ± 5 min. *Ad libitum* water intake was allowed after the 120 min blood draw and at Day 0, intake was recorded and replicated at Day 7. Immediately following the 240 min blood sample a standard lunch was consumed (see details above). At the end of each visit, the venous catheter was removed, and adverse events were assessed by an open-ended question. The investigator graded the adverse events by severity (mild, moderate, severe) and judged the likelihood they were related to the study product (definitely, probably, possibly, unlikely, not related). On Day 0, study product for at home consumption was dispensed, along with a Study Product Log and subjects were reminded of study instructions. Subjects were asked to return the Study Product Log and any unused study product at Day 7, to allow assessment of protocol compliance.

At home, subjects were instructed to consume their allocated daily SS of study product, split into two equal servings (see details above), and to record intake in the Study Product Log.

#### 2.2.4 Blood sample analysis

Analysis of blood samples collected during screening (Visit 1, Day −7) took place at Elmhurst Memorial Reference Laboratory (Elmhurst, IL). The clinical chemistry profile included, albumin, aspartate aminotransferase, alanine aminotransferase, alkaline phosphatase, total bilirubin, calcium, chloride, creatinine, blood urea nitrogen, potassium, sodium, total protein, carbon dioxide, osmolality and glucose. The hematology profile included white blood cell count, red blood cell count, hemoglobin concentration, hematocrit (as volume percent), mean corpuscular volume, mean corpuscular hemoglobin concentration, neutrophils, lymphocytes, monocytes, eosinophils, basophils and platelet count.

#### 2.2.5 Bioanalytic methods

Blood samples from PK visits at Day 0 and Day 7 were frozen until they were analyzed for BHB, BDO and HEX at Keystone Bioanalytical Inc. (North Wales, PA, United States).

BH-BD, BHB, BDO, and HEX were determined using high performance liquid chromatography/mass spectrometry (HPLC/MS) using gradient elution. For the *in vitro* plasma hydrolysis experiments, a Shimadzu LC-20AD binary HPLC connected with a Phenomenex Kinetex XB-C18 column, 50 × 2.1 mm, 2.6 µm and Sciex API5500 LC/MS/MS detector were used for the determination of BH-BD. Mass transitions under the positive with ESI (electrospray ionization) are m/z 287.2->171 and m/z 290.2 ->174 were used to monitor BH-BD and BH-BD -d3. Area ratio of the BH-BD to BH-BD -d3 was used for the quantitative determination of BH-BD with a calibration range 312.5 to 20,000 ng/mL. The HPLC flow was 0.5 mL/min using 0.1% formic acid in water, and 0.1% formic acid in acetonitrile (ACN) as the mobile phase with a gradient program; run time was 5 min. The BH-BD concentration at baseline (0 min) was used as 100% to calculate the rate of hydrolysis. Samples were analyzed in triplicate.

For the clinical study, PBS (Phosphate Buffered Saline, pH 7.4) was used as surrogate matrix for blanks, calibration standards and QCs preparation. Calibration standards and QCs were prepared by spiking the target analyte (BHB, BDO, HEX) into PBS. Standards used for calibration were as follows. For BHB, the reference standard was (±)-β-hydroxybutyrate (Cayman Chemical) and the internal standard was (±)-β-hydroxybutyrate-d4 (Cayman Chemical). For BDO, the reference standard was (±)-1,3-butanediol (Sigma-Aldrich) and the internal standard was (±)-1,3-butanediol-1,3–^13^C_2_ (Sigma-Aldrich). For HEX, the reference standard was hexanoic acid (Sigma-Aldrich) and the internal standard was hexanoic acid-d11 (Cayman Chemical). PBS was spiked with the appropriate concentrations of BHB, BDO, HEX to give target concentrations yielding a lower limit of quantitation (LLOQ) and upper limit of quantitation (ULOQ) of 0.5 μg/mL and 350 μg/mL, respectively, for BHB; 0.2 μg/mL and 150 μg/mL, respectively, for BDO; 0.1 μg/mL and 50 μg/mL, respectively, for HEX. Plasma samples were thawed and mixed by vortex. For BHB and BDO, 50 μL of plasma was combined with 50 μL of 50% ACN/50% water. To this, 50 μL internal standard solution (for BHB: 5 ug/mL of BHB-d4 in 50%ACN/50%water, for BDO: 5 ug/mL of DBO-d2 in 50%ACN/50%Water) was added. Finally, 0.15 mL ACN was added, the mixture vortexed, and centrifuged at 14,000 rpm and 4°C for 5 min. For analysis by HPLC, 25 μL of supernatant was combined with 225 μL nanopure water prior to injection. Sample preparation for HEX was similar. Internal standard solution (5 ug/mL of HEX-d11 in 50%ACN/50% water) was added to the 50:50 plasma: ACN/water and well-mixed. Centrifugation under the same conditions as used for BHB and BDO was followed by transfer of 50 μL supernatant into a 1.5 mL conical plastic tube. To this, 50 μL of 3-NPH (3-nitrophenylhydrazine), 50 μL EDAC (1-ethyl-3-3(3-dimethylaminopropyl)carbodiimide), and 50 μL of 10% pyridine solution was added, vortexed. Tubes were heated at 65°C for 15 min and then prepared for HPLC injection as described for BHB and BDO.

BHB, BDO, and HEX were determined by HPLC/MS using gradient elution. Two instrument systems were used for the analyses. System A for BHB and HEX comprised Shimadzu LC-20AD gradient pump, Shimadzu SIL-30AC autosampler, Shimadzu CTO-20AC column oven, Phenomenex Kinetex 2.6 µm XB-C18, 100A, 50 × 2 mm column, and AB Sciex QTrap 5500 detector. System B comprised Sciex AC gradient pump, Sciex AC Autosampler (AUS-14), Sciex AC column oven, and AB Sciex Triple Quad 5500 detector. System B used a Phenomenex Kinetex 2.6 µm XB-C18, 100A, 50 × 2 mm column for BHB and HEX, or a Phenomenex Kinetex 2.6 µm, C18, 100A 75 × 4.6 mm column for BDO. Detectors used an ESI (Electrospray ionization) interface in negative ion mode. For all systems/analytes, flow rate was 0.5 mL/min and injection volume was 1–2 uL. Eluent gradients are listed in Table 1. Column temperature was 40°C for HEX and 30°C for BDO and BHB. Mass transitions m/z 250.2->137.1 and m/z 261.2->137.1 were used to monitor HEX and HEX-d11. Mass transitions m/z 238.2->137.1 and m/z 242.2->137.1 were used to monitor BHB and BHB-d4. Mass transitions m/z 91->55 and m/z 93->57 were used to monitor BDO and BDO-d4. Area ratios of the species to the deuterated species were used for quantification.

**TABLE 1:** Gradient elution programs for systems A and B.

Min	HEX %B	BHB %B	Min	BDO %B
3	35	35	2.5	5
3.1	98	98	3.5	98
4.1	98	98	4.5	98
4.2	5	5	5.5	2.5
5	stop	stop	8	stop

Eluent A—0.1% formic acid in water, Eluent B–ACN (Acetonitrile).

### 2.3 Statistical methods

#### 2.3.1 Sample size

No formal sample size calculation was performed as this was a pilot study. The attrition rate was estimated to be ∼15%. Thus, this study included a sample size of 33 subjects to have 30 evaluable completers, with the option of over-enrollment in the event of early terminations. With 30 subjects (∼10 per group), there would be approximately 80% power to detect an effect size of 0.60 at a two-sided 0.05 significance level with a 1-way ANOVA.

#### 2.3.2 Analysis and endpoints

Concentrations at or below the quantification limit (QL) were observed for HEX and DB and were replaced with the QL divided by the square root of 2. The LLOQ for BDO and HEX was 0.2 and 0.1 μg/mL, respectively.

R version 4.2.0 with the package “NonCompart” (Bae, 2022) was used to calculate the non-compartmental analysis parameters for HEX, BDO, and BHB for each subject at each time point (Day 0 and Day 7). The following parameters were defined: Total AUC from pre-product consumption (t = −0.25 h) to 8 h (AUC_0–8h_) where the time prior to product consumption (t = −15 min) was used as time 0. The AUC was calculated by the linear-log trapezoidal method (“linear-up log-down”) where the linear trapezoidal method was used when concentrations were increasing (“absorption phase”) and the logarithmic trapezoidal method used when the concentrations were decreasing (“elimination phase”).

Maximal concentration (C_max_) was defined as the maximum recorded concentration over the 8 h collection period and time of maximal concentration (T_max_) was defined as the time of the maximum concentration. Half-life (t_1/2_) was calculated as the natural log of 2 divided by the estimate of the terminal elimination rate constant (λ_Z_) where λ_Z_ was estimated by negative slope of the linear regression of the terminal data for the time *versus* log concentration curve.

#### 2.3.3 Outcome analysis

All tests of significance are two-sided and considered significant at the 0.05 level. All analyses were performed on the mITT and the PP population where the mITT population served as the primary analysis population.

Acute and chronic response were represented by PK results measured at Day 0 and Day 7, respectively. For each outcome, a linear mixed model was used to estimate the between group comparisons along with the corresponding 95% confidence interval at each measured day. Fixed effect terms included time point (i.e., Day 0, Day 7), group, and the time point by group interaction. Between-group comparisons at each time point were evaluated with the overall F-test with contrasts statements. If significant at the 0.10 level (at least marginally significant), then all respective pairwise comparisons were performed (Group 1 vs. Group 2, Group 1 vs. Group 3, and Group 2 vs. Group 3). Pairwise comparisons were evaluated at the 0.05 significance level and the mean difference along with the corresponding 95% confidence interval were estimated. A log transformation was considered for the half-life estimation for BHB and HEX; back transformed results were provided.

#### 2.3.4 Post hoc analysis

A subgroup analysis was performed to evaluate the impact of the dosage per body weight within each intervention group. Dosage per body weight (g/kg) was calculated as the assigned g of BH-BD divided by the subject weight captured at screening (kg). The respective PK parameters were then estimated at each study day along with a 95% confidence interval. Additionally, the dose-normalized AUC and C_max_ calculated for each subject was summarized across groups as mean (standard deviation [SD]) and median (interquartile range [IQR] limits).

## 3 Results

### 3.1 *In vitro* BH-BD hydrolysis

**FIGURE 3:**
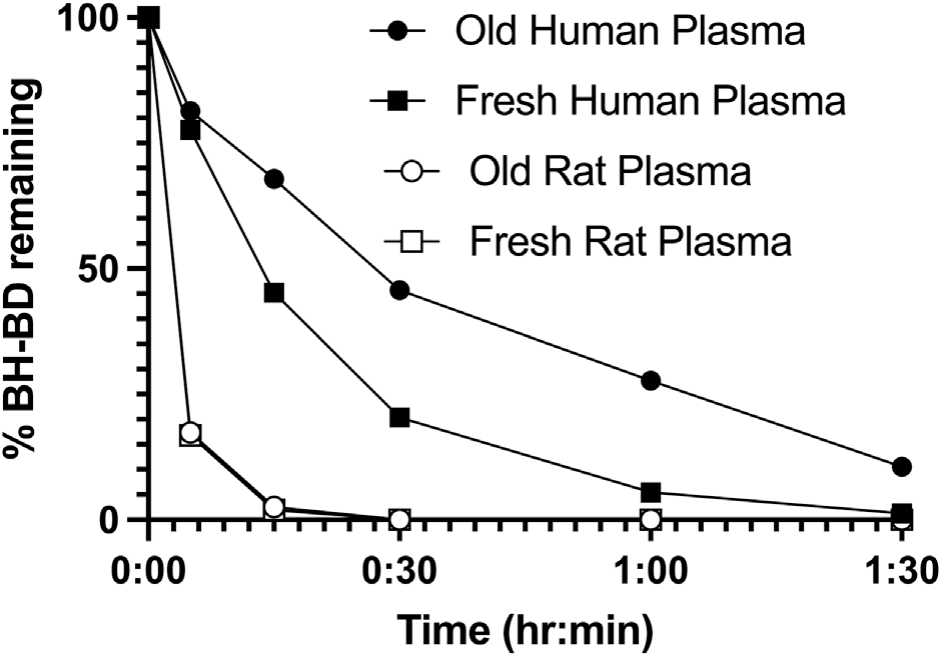
Percentage of intact BH-BD (20 mg/mL) remaining over time when incubated with fresh or old human or rat plasma (5 mL).

BH-BD incubated with both fresh and old (previously frozen) human and rat plasma displayed rapid degradation within 1.5 h (Figure 3). Degradation of BH-BD was equally fast in both fresh and old rat plasma, reaching 0% remaining within 30 min. Human plasma incubation resulted in comparatively slower BH-BD hydrolysis compared to rat. BH-BD was broken down more quickly in fresh compared to old (previously frozen) human plasma (Fresh = < 50% in 15 min vs. Old = < 50% in 30 min), and a small amount (10.6%) remained in old human plasma after 1.5 h incubation. Nonetheless, this data demonstrated that esterase enzymes in human plasma rapidly degrade intact BH-BD, if any were to reach the plasma after oral ingestion.

### 3.2 Clinical study population

**FIGURE 4:**
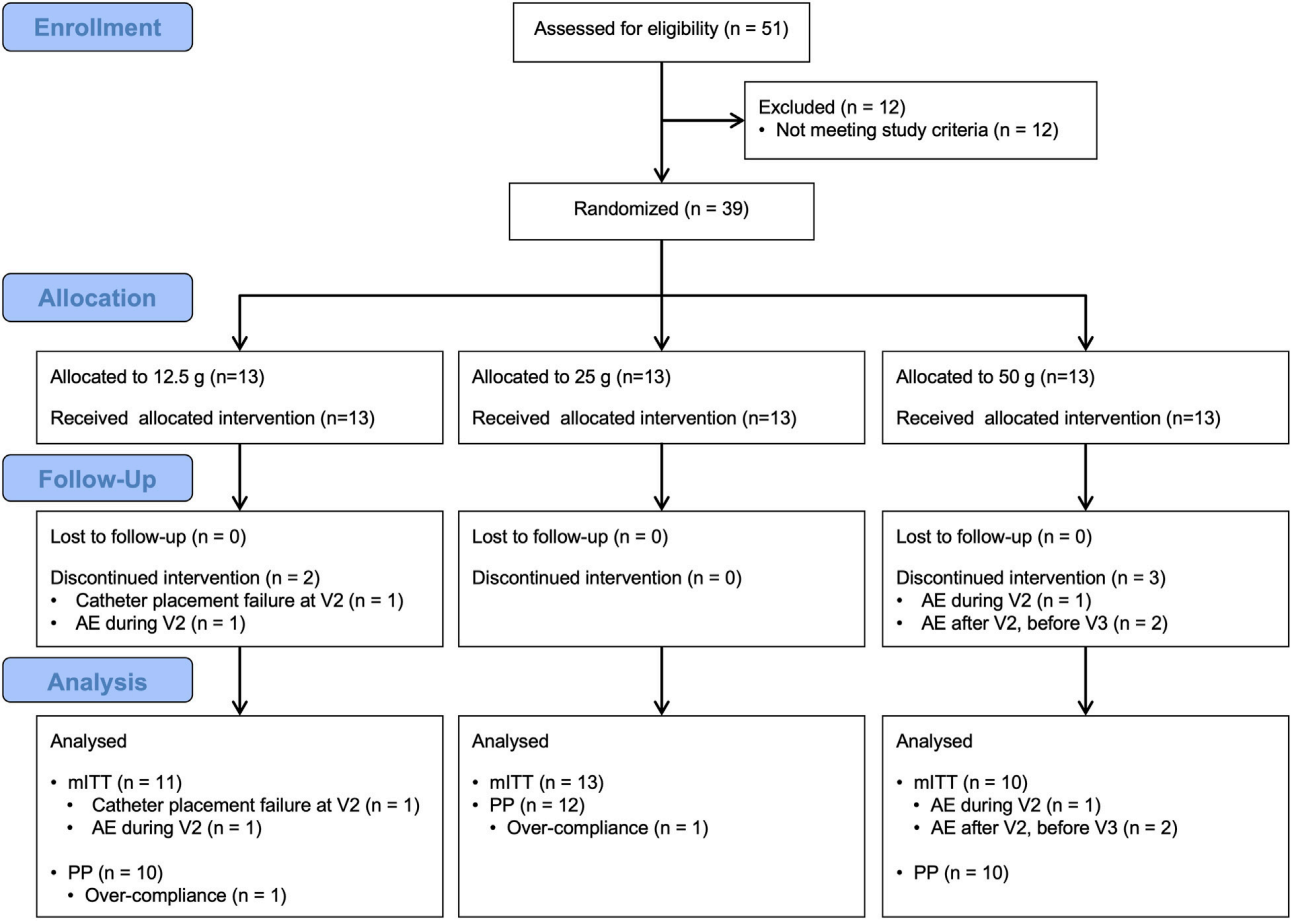
Study flow chart showing the disposition of subjects by study group. Abbreviations: mITT: modified intent to treat, PP: per protocol.

Subject characteristics are shown in Table 2. The mITT population consisted of 34 subjects who provided as least one analyzed post-baseline data point. The PP population consisted of 32 subjects (Figure 4). All subjects consumed the assigned amount of study beverage during the clinic visits. For at home consumptions, beverage compliance was consistent across group. The median compliance was 100% for each beverage group. The mean was 106.1% (SD 15.4), 105.1% (SD 10.5), and 102.5% (SD 5.6) for the 12.5, 25, and 50 g groups, respectively. In the PP population, the mean was 101.2% (SD 5.3), 102.8 (SD 6.5), and 102.5% (SD 5.6) for the 12.5, 25, and 50 g groups, respectively. There were no qualitative differences between the results for the mITT and PP populations; thus, only the results for the mITT population are shown in this manuscript.

**TABLE 2:** Clinical study subject characteristics. Abbreviations: BMI: body mass index, IQR: interquartile range, n: sample size, SD: standard deviation.

Characteristic	Statistic/Category	Overall	12.5 g	25 g	50 g
Sex	Female	18 (52.9%)	5 (45.5%)	7 (53.8%)	6 (60.0%)
	Male	16 (47.1%)	6 (54.5%)	6 (46.2%)	4 (40.0%)
BMI (kg/m^2^)	n	34	11	13	10
	Median (Range)	27.7 (19.0, 34.5)	28.4 (19.0, 34.4)	27.1 (22.5, 33.6)	27.1 (23.0, 34.5)
	IQR Limits	25.0, 32.9	21.0, 33.3	25.0, 32.9	26.6, 30.1
	Mean (SD)	28.0 (4.5)	28.0 (5.7)	28.2 (4.3)	27.8 (3.5)
Age (years)		34	11	13	10
	Median (Range)	47.5 (20.0, 65.0)	51.0 (21.0, 64.0)	41.0 (20.0, 65.0)	47.0 (29.0, 64.0)
	IQR Limits	37.0, 55.0	44.0, 60.0	35.0, 62.0	37.0, 53.0
	Mean (SD)	46.4 (12.9)	49.8 (12.0)	44.4 (15.1)	45.3 (11.2)
Race	Black/African American	5 (14.7%)	3 (27.3%)	0 (0%)	2 (20.0%)
	Multiracial	1 (2.9%)	0 (0%)	1 (7.7%)	0 (0%)
	White	28 (82.4%)	8 (72.7%)	12 (92.3%)	8 (80.0%)
Ethnicity	Hispanic/Latino	6 (17.6%)	1 (9.1%)	4 (30.8%)	1 (10.0%)
	Not Hispanic/Latino	28 (82.4%)	10 (90.9%)	9 (69.2%)	9 (90.0%)
Weight (kg)		34	11	13	10
	Median (Range)	82.1 (54.1, 111.3)	88.4 (54.1, 108.8)	78.1 (61.4, 111.3)	82.6 (63.0, 98.3)
	IQR Limits	75.5, 89.9	57.1, 101.0	75.6, 88.6	76.7, 88.5
	Mean (SD)	81.9 (14.7)	83.3 (20.1)	80.5 (12.8)	82.3 (11.2)

### 3.3 Plasma beta hydroxybutyrate

**FIGURE 5:**
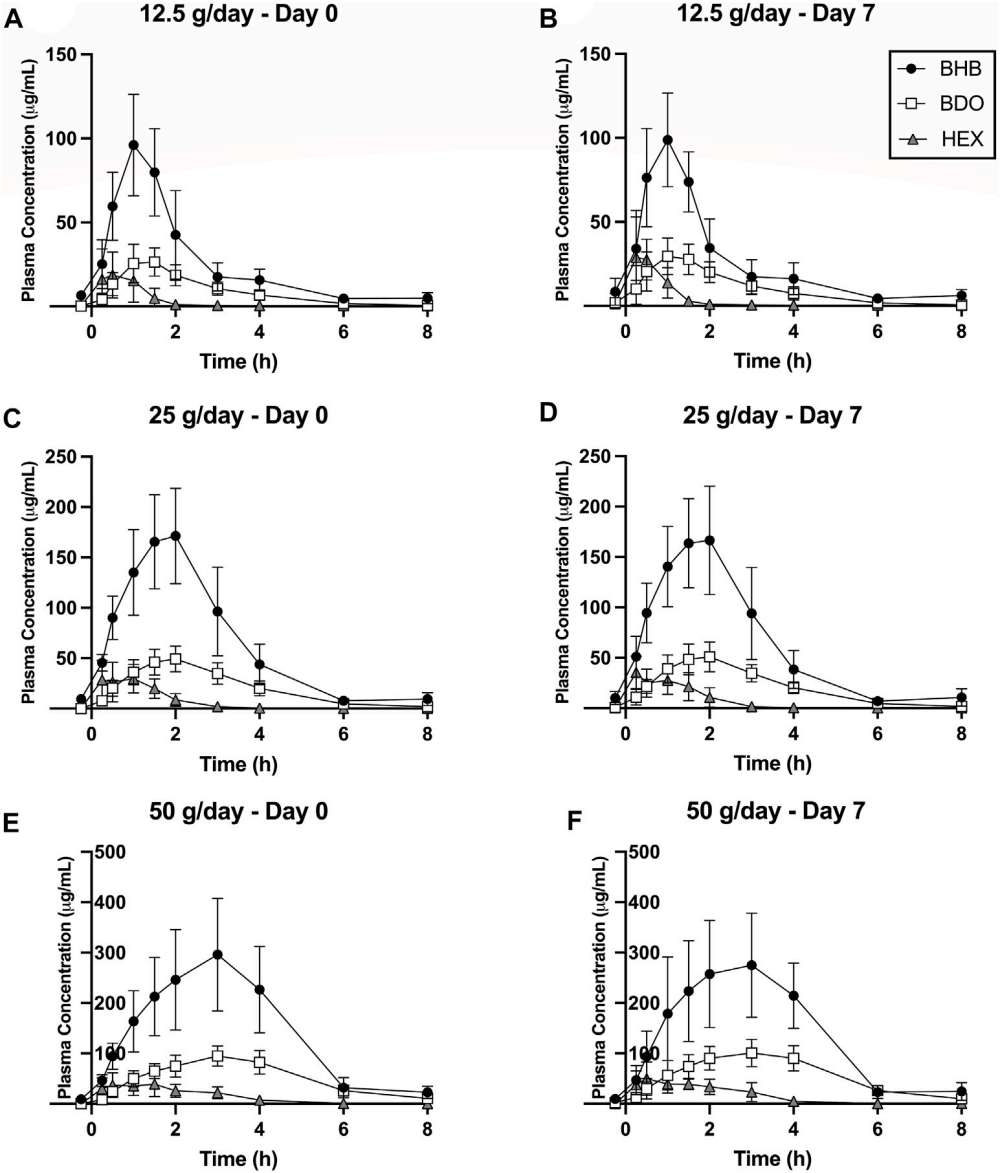
Time course of BH-BD metabolites, BHB, BDO and HEX in healthy adults consuming 12.5 g (*n* = 11), 25 g (*n* = 13) and 50 g (*n* = 10) in the naïve state [Day 0: **(A, C, E)**] and after a week of daily consumption [Day 7: **(B, D, F)**]. Data are mean (SD). Abbreviations: BHB: beta hydroxybutyrate, BDO: (R)-1,3-butanediol, HEX: hexanoic acid.

**FIGURE 6:**
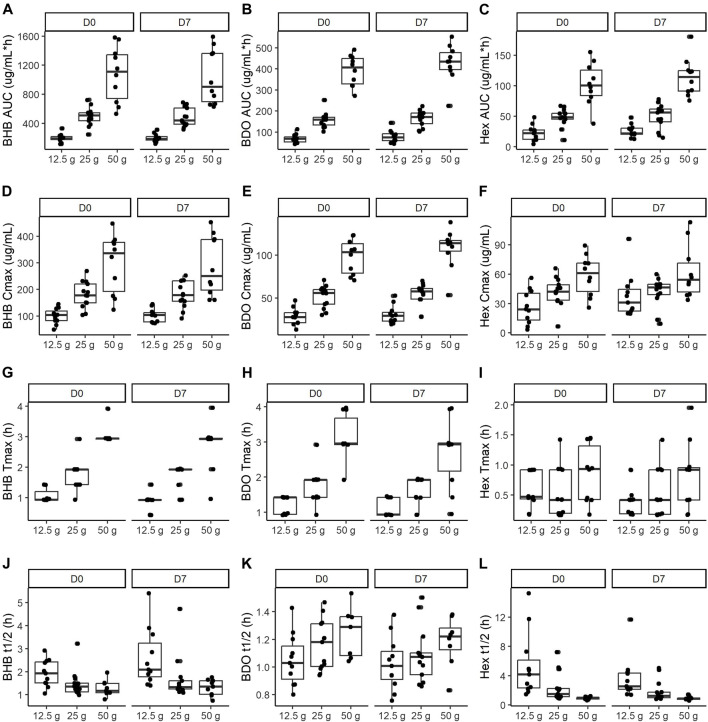
Kinetic parameters of BH-BD metabolites: BHB **(A, D, G, J)**, BDO **(B, E, H, K)** and HEX **(C, F, I, L)** in healthy adults consuming 12.5 g (*n* = 11), 25 g (*n* = 13) and 50 g (*n* = 10) in the naïve state (Day 0) and after a week of daily consumption (Day 7). Data are median (IQR) Abbreviations: AUC: area under the curve, BHB: beta hydroxybutyrate, BDO: (R)-1,3-butanediol, C_max_: maximal concentration, t_1/2_: half-life, HEX: hexanoic acid, T_max_: time of maximal concentration.

Blood BHB concentration increased immediately following consumption of all serving sizes of BH-BD, on both study days and consistently returned to baseline by the end of the study Figures 5A–F. BHB concentration was consistently higher than BDO and HEX at all time points within each dose group. BHB concentrations in ug/mL were converted to the common unit (mM), this is shown in the Supplementary Material S1 (Supplementary Figure S7). BHB AUC, C_max_ and T_max_ all increased approximately in proportion to SS whereas t_1/2_ for BHB and HEX tended to decrease with increasing SS (Figures 6A, D, G, J; Table 3). There was a consistent strong effect of group on all kinetic parameters of BHB but no day*group interaction (Table 4). Additionally, *post hoc* subgroup analysis suggested that body weight was not a contributing factor to the variation for each PK outcome within each intervention group.

**TABLE 3:** Kinetic parameters of BH-BD metabolites in healthy adults consuming 12.5 g (*n* = 11), 25 g (*n* = 13) and 50 g (*n* = 10) in the naïve state (Day 0) and after a week of daily consumption (Day 7). Data are mean (SD). Significance was taken at *p* < 0.05 and is shown as^a^ 12.5 vs. 25 g,^b^ 12.5 vs. 50 g and^c^ 25 vs. 50 g. For BHB, one subject did not have enough data points after the measured maximum concentration at Day 0 and Day 7 to estimate the half-life. For BD, three subjects at Day 0 and two subjects at Day 17 had insufficient data to estimate the half-life. Abbreviations: AUC: area under the curve, BHB: beta hydroxybutyrate, BDO: (R)-1,3-butanediol, C_max_: maximal concentration, t_1/2_: half-life, HEX: hexanoic acid, T_max_: time of maximal concentration.

	C_max_ (ug/mL)		AUC (ug/mL*h)		T_max_ (h)		t_1/2_ (h)	
	Day 0	Day 7	Day 0	Day 7	Day 0	Day 7	Day 0	Day 7
BHB—12.5 g	99.4^a,b^ (28.8)	102.4^a,b^ (25)	196.8^a,b^ (58.3)	197.8^a,b^ (56.5)	1.1^a,b^ (0.2)	0.9^a,b^ (0.3)	1.9^b^ (0.6)	2.6^a,b^ (1.2)
BHB—25 g	179.6^c^ (49.6)	179.9^c^ (50)	491.2^c^ (124.8)	481.6^c^ (127.8)	1.8^c^ (0.5)	1.7^c^ (0.4)	1.5 (0.6)	1.7 (1)
BHB—50 g	296.2 (111.4)	286.9 (112.1)	1076.3 (381.4)	1034.4 (376.8)	3 (0.3)	2.7 (0.8)	1.3 (0.3)	1.3 (0.3)
BDO—12.5 g	28.7^a,b^ (9.7)	30.7^a,b^ (10.5)	68.5^a,b^ (21.1)	78.2^a,b^ (29)	1.2^a,b^ (0.3)	1.1^a,b^ (0.3)	1.1^b^ (0.2)	1 (0.2)
BDO—25 g	51.9^c^ (12.9)	54.2^c^ (13)	158.5^c^ (37.1)	163.6^c^ (38.3)	1.8^c^ (0.5)	1.7^c^ (0.4)	1.2 (0.2)	1.1 (0.2)
BDO—50 g	97.9 (20)	107.6 (23)	391.9 (73)	427 (89.8)	3.1 (0.6)	2.7 (1)	1.3 (0.2)	1.2 (0.2)
HEX—12.5 g	26.7^b^ (17.3)	37.8^b^ (22.6)	22.2^a,b^ (13)	25.9^a,b^ (10.6)	0.6 (0.3)	0.4^b^ (0.2)	5.4^a,b^ (4.4)	3.7^a,b^ (2.9)
HEX—25 g	41.9^c^ (14.6)	41.6^c^ (15)	48.3^c^ (15.3)	49.2^c^ (20.3)	0.5^c^ (0.4)	0.5 (0.4)	2.4^c^ (2)	1.9^c^ (1.5)
HEX—50 g	58.7 (20.6)	62 (27)	102.5 (34.3)	114.1 (31.1)	0.9 (0.5)	0.8 (0.6)	0.9 (0.2)	0.9 (0.3)

**TABLE 4:** Significance of type 3 fixed effects in linear model of BH-BD metabolite kinetic parameters with 3 serving size groups (12.5, 25 and 50 g) on 2 days (Day 0 and Day 7). Abbreviations: AUC: area under the curve, BHB: beta hydroxybutyrate, BDO: (R)-1,3-butanediol, C_max_: maximal concentration, t_1/2_: half-life, HEX: hexanoic acid, SS: serving size, T_max_: time of maximal concentration.

		Type 3 fixed effects		
		Day	SS Group	Day* SS Group
BHB	AUC	0.49	<.001	0.768
	C_max_	0.82	<.001	0.839
	T_max_	0.058	<.001	0.472
	t_1/2_	0.052	0.001	0.341
BDO	AUC	0.014	<.001	0.144
	C_max_	0.072	<.001	0.4
	T_max_	0.031	<.001	0.282
	t_1/2_	0.129	0.061	0.843
HEX	AUC	0.1	<.001	0.391
	C_max_	0.241	0.001	0.474
	T_max_	0.303	0.031	0.521
	t_1/2_	0.163	<.001	0.663

### 3.4 Plasma (R)-1,3-butanediol

Blood BDO concentration increased immediately following consumption of all serving sizes of BH-BD, on both study days and consistently returned to baseline by the end of the study Figures 5A–F. BDO concentration was consistently lower than BHB and tended to remain elevated for longer than HEX within each dose group. BDO AUC, C_max_ and T_max_ all increased with increasing SS, whereas t_1/2_ was generally consistent across different SS (BDO t_1/2_ Day 0, *p* = 0.077; Day 7, *p* = 0.199) (Figures 6B, E, H, K; Table 3). There was a consistent strong effect of group on AUC, C_max_ and T_max_, but not on t_1/2_. There were no significant day*group interactions (Table 4). Additionally, *post hoc* subgroup analysis suggested that body weight was not a contributing factor to the variation for each PK outcome within each intervention group.

### 3.5 Plasma hexanoic acid

Blood HEX concentration increased immediately following consumption of all serving sizes of BH-BD, on both study days and consistently returned to baseline by at least 4 h after ingestion Figures 5A–F. HEX concentration was consistently lower than BHB and BDO; tending to return to baseline more rapidly than the other metabolites within each dose group. HEX AUC and C_max_ tended to increase with increasing SS, whereas t_1/2_ consistently decreased with increasing SS (Figures 6C, F, I, L; Table 3). Overall F-test was significant for T_max_ at Day 7 (*p* = 0.037), but not at Day 0 (*p* = 0.109). Pairwise comparisons performed at Day 7 indicate that HEX T_max_ was significantly shorter following 12.5 g compared to 50 g (*p* = 0.012). There was a consistent fixed effect of group on all kinetic parameters, but no significant effect of day, or day*group interactions (Table 4). Additionally, *post hoc* subgroup analysis suggested that body weight was not a contributing factor to the variation for each PK outcome within each intervention group.

### 3.6 Adverse events

A total of thirteen subjects reported adverse events (AEs) at least once during the 8 days of the study. A majority of these (25/31) were gastrointestinal (i.e., abdominal cramping/bloating, stomachache/cramps, heartburn, nausea, loose stools, diarrhea, constipation, appetite suppression, and vomiting). Other AEs included headache, light-headedness, dizziness, visual changes, and fatigue. Of the AEs reported, 23/31 were judged to be definitely or probably related to the study product. In four subjects (*n* = 3 in the 50 g SS group, *n* = 1 in the 12.5 g SS group), side effects occurred on the first PK Visit (Visit 2) that led to an early termination of these subjects. All AEs were judged as mild or moderate in severity (none were severe) and were confirmed to have resolved during follow-up.

## 4 Discussion

This study aimed to investigate the fate of the novel ketone ester BH-BD and the PK of its hydrolysis and metabolic products, BHB, BDO and HEX in healthy adults across a range of doses and following a week of consumption. The main findings are that BH-BD was rapidly hydrolysed in human plasma and its consumption in healthy adults resulted in serving size dependant elevations in blood concentrations of BHB, with smaller increases in BDO and HEX. Furthermore, there were no consistent changes to PK parameters of any metabolites after 7 days of daily BH-BD consumption, indicating a lack of saturation or adaption to BH-BD metabolism in this time frame.

Whilst previous studies had determined the rapid (∼15 min), complete *in vitro* hydrolysis of BH-BD in rat plasma, gastric, intestinal, liver and caecal model systems (Stubbs et al., 2021), it was unknown if human model systems would give similar results. Given that humans consuming BH-BD exhibited similar plasma BHB concentrations to rats at far lower doses (human = 300 mg/kg, 0.8 mM, rat = 1,500 mg/kg, 0.8 mM) (Stubbs et al., 2021; Crabtree et al., 2022), we initially suspected that humans may exhibit more effective breakdown of BH-BD than rats. Here we found that complete *in vitro* hydrolysis of BH-BD in human plasma was in fact slower than in rat plasma (∼30 min). This suggests that greater hepatic conversion of BDO and HEX to BHB underlies the relatively stronger ketogenic response in humans compared to rats. In support of this, rats display markedly lower blood ketone concentrations during ketoacidosis compared to humans (rat: ∼3 mM, human ∼8 mM) (Thompson et al., 1994; Kitabchi et al., 2008). Nonetheless, over 50% of BH-BD was hydrolysed within 15 min of incubation in fresh human plasma, so when taken with our previous finding that no intact BH-BD was detected in rodents administered 12,000 mg/kg of BH-BD, the data suggests that intact BH-BD is unlikely to be present in high concentrations in human plasma following BH-BD consumption.

The data from our human PK study further supports that BH-BD and its hydrolysis products are efficiently converted into ketone bodies (i.e., BHB) in humans. Whilst plasma concentrations of BH-BD itself were not measured, we observed SS dependant increases in plasma BHB concentrations, with relatively lower circulating concentrations of the direct hydrolysis products BDO and HEX. The BH-BD molecule consists of two molecules of HEX each esterified to a central BDO molecule and there is no BHB directly generated as a result of ester bond hydrolysis. Based on the mass of BDO and HEX administered at each SS and the mass that was recovered in the plasma (AUC), we calculated that approximately 20%–30% of administered BDO appeared in plasma, compared to ∼3% for HEX (data not shown). This indicates a high removal of HEX either by conversion to BHB or into alternative lipid metabolism pathways; tracer experiments would be required to confirm the proportional fate of HEX. Previous tracer studies of BDO indicated a high (85%—98%) conversion to BHB (Desrochers et al., 1992). Taken together, this supports that the major metabolic fate of BH-BD, and its hydrolysis products, is efficient conversion to ketone bodies.

This study both supports and extends previous PK studies of BH-BD, which administered 12.5 and 25 g serving sizes and found a proportional increase in blood BHB concentrations at these doses. The 12.5 and 25 g SS groups in this study exhibited very similar BHB C_max_ (Day 0: 12.5 g = 1.0 mM, 25 g = 1.7 mM) to studies of BH-BD beverages in healthy adults consumed with a meal by Crabtree *et al* (12.5 g = 0.8 mM, 25 g = 1.7 mM) (Crabtree et al., 2022) and Nieman *et al* (12.5 g = 0.9 mM) (Nieman et al., 2022). This high replicability between study populations provides confidence in dosing selection for future studies using BH-BD in similar populations. Additionally, this study extends the understanding of the dose: response relationship by including a 50 g SS group. The PK parameters of BHB delivery (AUC and C_max_) increased proportionally across the 12.5—50 g SS range studied, in a similar manner as seen with other ketone esters (Shivva et al., 2016). Overall, these results demonstrate that modification of BH-BD SS between 12.5—50 g can titrate the degree of nutritional ketosis between 0.8–2.8 mM, making BH-BD a useful tool if a specific BHB concentration were desired to achieve a functional endpoint. Emerging research has begun to identify optimal BHB levels for several benefits such that increases in BHB lead to greater cognition, cardiac function, and sports performance (Nielsen et al., 2019; Fortier et al., 2021; Evans et al., 2022), although this is still poorly defined. Additionally useful in this regard is the observation that body mass index did not significantly impact the dose response to BH-BD. This is an important observation that corroborates the safe use of BH-BD in foodstuffs. Foods are not typically designed to deliver a substance based on body weight.

No consistent changes in PK parameters occurred following 1 week of daily BH-BD ingestion, indicating that saturation of, or adaptation to BH-BD metabolism did not occur at these SS within this time period. This is in keeping with previous data using exogenous ketones, which found that there were no changes in BHB C_max_ after daily consumption of ketone ester drinks for three to 4 weeks in healthy adults (Soto-Mota et al., 2019; Chen et al., 2021), diabetics (Soto‐Mota et al., 2021) and athletes (Poffe et al., 2019). We did note an apparent dose dependent decrease in t_1/2_ for BHB and HEX, but not for BDO, on both study days. BHB and HEX are unique from BDO in that their removal is driven by direct oxidation for ATP formation, in a manner that is likely to be concentration dependant within the “physiological” range seen in this study. For example, the brain will oxidize BHB in proportion to the level that circulates in plasma (Cuenoud et al., 2020). Furthermore, carnitine transport is not required for clearance and metabolism of HEX and it is known that the liver rapidly oxidizes short and medium chain fatty acids if they are available as opposed to storing them in adipose (Schonfeld and Wojtczak, 2016). By contrast, BDO is cleared from circulation by the alcohol metabolism pathway in the liver, largely to form BHB (Desrochers et al., 1992). The kinetics of this specific conversion are poorly defined (Tate et al., 1971), and may exhibit distinct kinetic parameters compared to substrate (BHB and HEX) removal via oxidation. Given this, we speculate that increasing concentration of BHB and HEX could result in relatively faster removal due to a relative increase in the utilization of BHB and HEX as source of metabolic energy.

This study has some limitations which should be considered when interpreting the results. Firstly, the 1-week daily consumption period may not have been sufficient to allow adaptive changes to occur, although previous studies using ketone esters do not indicate this would be the case (Poffe et al., 2019; Soto-Mota et al., 2019; Chen et al., 2021; Soto‐Mota et al., 2021). Secondly, as this was a parallel group, non-crossover study, inter-individual differences could not be eliminated. Thirdly, we did not measure intact BH-BD in the human PK study, which would have confirmed the lack of systemic exposure suspected based on the *in vitro* data. We also did not measure the secondary blood ketone body, acetoacetate, which would have provided a more complete picture of the ketogenic effect of BH-BD. Finally, as this was a pilot study, no power calculation was performed and the sample size was small, however given the close replication of previous PK studies using BH-BD, these results are likely to portray an accurate picture.

## 5 Conclusion

These results demonstrate that BH-BD is rapidly hydrolysed by humans and the hydrolysis products BDO and HEX are converted into the ketone body BHB in a dose dependant manner, at servings of up to 50 g. A relatively small amount of BDO and HEX reaches the systemic circulation but is cleared within hours of BH-BD consumption. There were no consistent changes to PK parameters of BHB, BDO or HEX that indicated adaptation to BH-BD consumption or metabolic saturation. This data supports the safety of BH-BD consumption in ketosis-promoting nutritional products.

## Data Availability

The raw data supporting the conclusion of this article will be made available by the authors, without undue reservation.

## Supplementary material

SUPPLEMENTARY FIGURE S1 BHB response by group in mM in plasma.
