# Supplementary figures and images for: A randomized, open-label, parallel pilot study investigating metabolic product kinetics of the novel ketone ester, bis-hexanoyl (R)-1,3-butanediol, over one week of ingestion in healthy adults
Source: Front Physiol. 2023 Jun 22;14:1196535. doi: 10.3389/fphys.2023.1196535 (PMC10324611; doi:10.3389/fphys.2023.1196535)

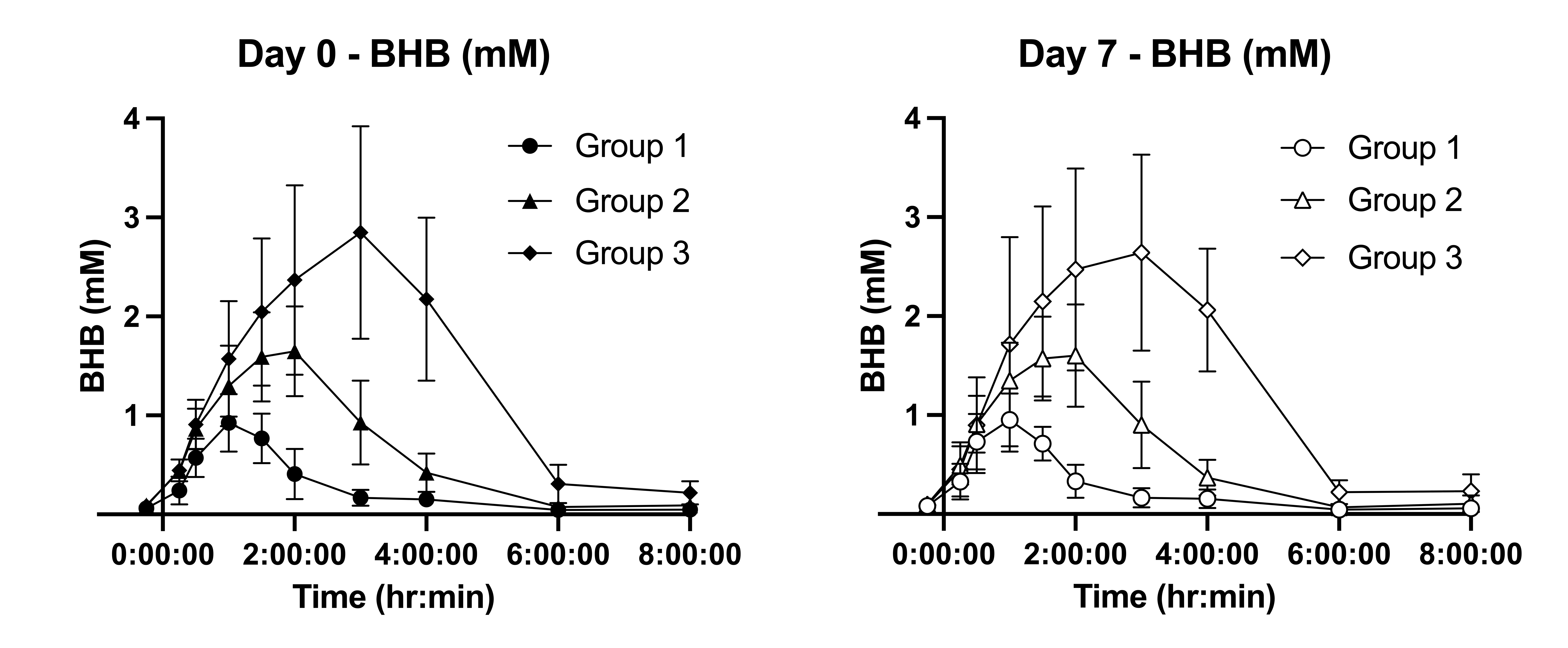

Supplement: Supplementary file 1 [file Image1.jpeg]
