# Supplementary material for: A randomized, open-label, parallel pilot study investigating metabolic product kinetics of the novel ketone ester, bis-hexanoyl (R)-1,3-butanediol, over one week of ingestion in healthy adults
Source: Front Physiol. 2023 Jun 22;14:1196535. doi: 10.3389/fphys.2023.1196535 (PMC10324611; doi:10.3389/fphys.2023.1196535)

**Supplemental Information**

**Table S5:** Full inclusion and exclusion criteria

### Inclusion Criteria

1. Subject is male or female, 18-65 years of age, inclusive at Visit 1 (Day -7).
2. Subject has a BMI 18.5-34.99 kg/m^2^ (inclusive) at Visit 1 (Day -7).
3. Subject is willing to consume the study beverage daily starting on Day 0 through Day 7 following taste testing at Visit 1 (Day -7).
4. Subject is not a smoker and has no plans to change his/her usage of the following products throughout the study period: tobacco, smoking products (including, but not limited to cigarettes, cigars, chewing tobacco, e-cigarettes), and nicotine products (e.g., nicotine gum and/or nicotine patches).
5. Subject is a non-user or former user (cessation ≥3 months) of any marijuana or hemp products and has no plans to use marijuana or hemp products during the study period. No washout is required for topical marijuana or hemp products, but subjects are required to abstain from these products during the study period.
6. Subject is willing and able to comply with all study procedures including overnight fasting (12 ± 2 h, water only), maintenance of usual body weight, avoidance of foods or supplements that increase ketone production in the body throughout the entire study period, avoidance of vigorous exercise (moderate habitual exercise is allowed) with the exception of the 24 h prior to Visits 2 and 3 (Days 0 and 7) when subjects must avoid exercise completely.
7. Subjects is willing to abstain from caffeine during Visits 2 and 3 (Days 0 and 7).
8. Subject has a score of 7 to 10 on the Vein Access Scale at Visit 1 (Day -7).
9. Subject is willing and able to comply with the visit schedule.
10. Subject has no health conditions that would prevent him/her from fulfilling the study requirements as judged by the Clinical Investigator on the basis of medical history, physical examination findings, and routine laboratory test results.
11. Subject understands the study procedures and signs forms providing informed consent to participate in the study and authorizes the release of relevant protected health information to the Clinical Investigator.

### Exclusion Criteria

1. Subject has a known allergy, intolerance, or sensitivity to any of the ingredients in the study beverages and all other foods/beverages provided in this study, including soy, milk, and peanut.
2. Subject has extreme dietary habits (e.g., intermittent fasting or time restricted eating, Atkins diet, vegan, very high protein/low carbohydrate within 30 days of Visit 1 (Day -7).
3. Subject has used weight-loss medications (including over-the-counter medications and/or supplements) or participated in weight loss programs within 30 days of Visit 1 (Day -7).
4. Unstable use of any prescription medications is not allowed. Prescription medications must be kept at a stable dose (defined as same dose for the past 30 days prior to Visit 1, Day -7)
5. Subject has used ketone supplements (ketone salts or esters, and medium chain triglycerides [MCT]) within 30 days of Visit 1 (Day -7).
6. Subject has been exposed to any non-registered drug product within 30 days of Visit 1 (Day -7).
7. Subject has a history or presence of clinically important endocrine (including hyperparathyroidism, type 1 or 2 diabetes mellitus and/or hypoglycemia), cardiovascular (including, but not limited to history of myocardial infarction, peripheral arterial disease, stroke), pulmonary (including uncontrolled asthma), hepatic, renal, gastrointestinal, hematologic, immunologic, dermatologic, rheumatic (including gout), and/or biliary condition(s), that, in the opinion of the Clinical Investigator (has MD qualifications), could interfere with the interpretation of the study results.
8. Subject has a history of bariatric surgery for weight reducing purposes
9. Subject has a history or presence of cancer in the prior two years, except for non-melanoma skin cancer.
10. Subject has an abnormal laboratory test result(s) of clinical importance at Visit 1 (Day -7), at the discretion of the Clinical Investigator. One re-test will be allowed on a separate day prior to Visit 2 (Day 0), for subjects with abnormal laboratory test results.
11. Subject has any signs or symptoms of an active infection of clinical relevance (e.g., urinary tract or respiratory) within 5 days of Visit 2 (Day 0). If an infection occurs during the study period, test visits should be rescheduled until all signs and symptoms have resolved (at the discretion of the Clinical Investigator) and any treatment (e.g., antibiotic therapy) has been completed at least 5 days prior to testing.
12. Subject has experienced any major trauma or any other surgical event within three months of Visit 1 (Day -7).
13. Subject has had a loss of 400 mL of blood (e.g., blood/plasma donation) within 30 days of Visit 2 (Day 0).
14. Subject has recently (within 6 months of Visit 1; Day -7) had a weight loss or gain >4.5 kg.
15. Subject has uncontrolled hypertension (systolic blood pressure ≥140 mm Hg or diastolic blood pressure ≥90 mm Hg) as defined by the blood pressure measured at Visit 1 (Day -7). One re-test will be allowed on a separate day before Visit 2 (Day 0), for subjects with abnormal blood pressure at the discretion of the Clinical Investigator.
16. Subject is a female who is pregnant, planning to be pregnant during the study period, lactating, or is of childbearing potential and is unwilling to commit to the use of a medically approved form of contraception throughout the study period. The method of contraception must be recorded.
17. Subject works nights or shifts that does not allow him/her to maintain a consistent meal schedule during the study.
18. Subject has a recent history of (within 12 months of screening; Visit 1; Day -7) or strong potential for alcohol or substance abuse. Alcohol abuse is defined as >14 drinks per week (1 drink = 12 oz beer, 5 oz wine, or 1½ oz distilled spirits).
19. Individual has a condition the Clinical Investigator believes would interfere with his/her ability to provide informed consent, comply with the study protocol, which might confound the interpretation of the study results, or put the subject at undue risk.

### Excluded Medications/Products and Foods

- Any marijuana or hemp products within 3 months of Visit 1 (Day -7) and throughout the study period
- Weight-loss medications (including over-the-counter medications and/or supplements) or participated in weight loss programs within 30 days of Visit 1 (Day -7)
- Ketone supplements (ketone salts or esters, and medium chain triglycerides [MCT]) within 30 days of Visit 1 (Day -7)
- Any non-registered drug product within 30 days of Visit 1 (Day -7)
- Unstable use of any prescription medications. Prescription medications must be kept at a stable dose (defined as same dose for the past 30 days prior to Visit 1, Day -7)
- Abstain from topical marijuana or hemp products during the study period

**Figure S7:** BHB response by group in mM in plasma.


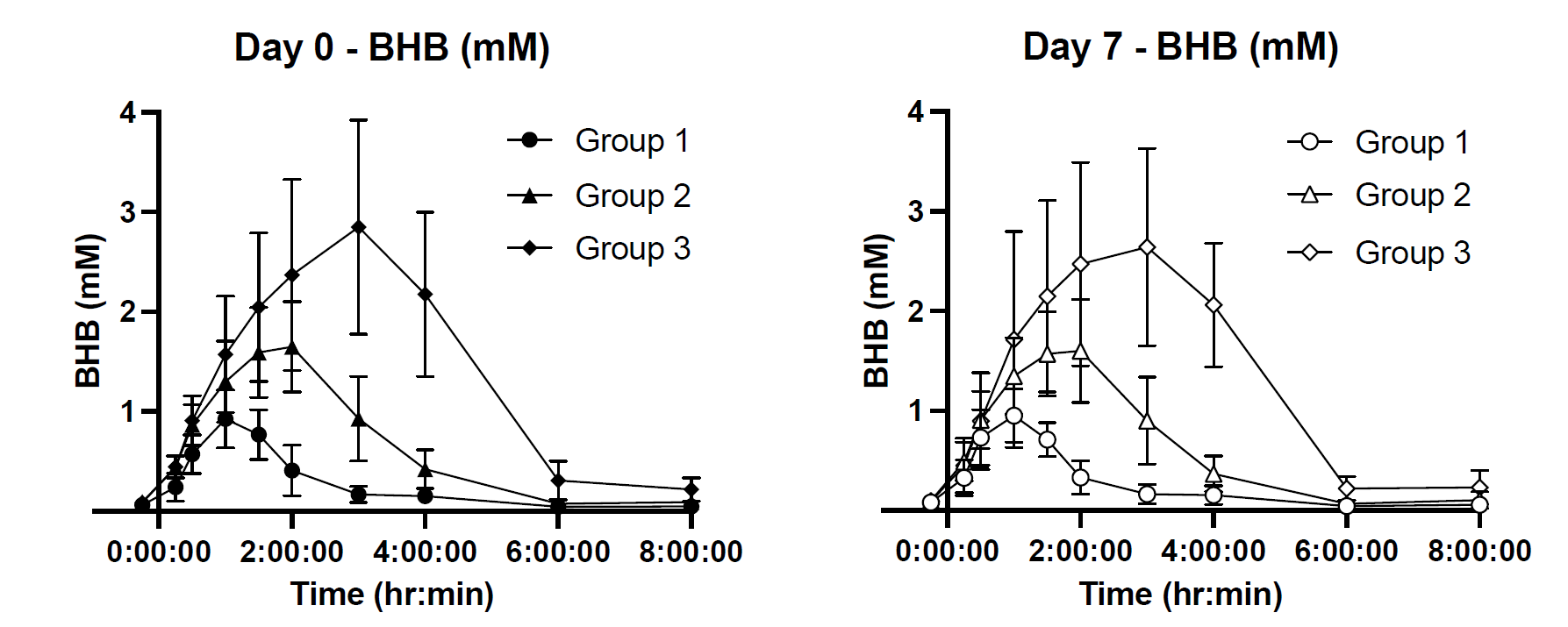

Supplement: Supplementary file 2 [file DataSheet1.docx]
